# PARP Inhibitors in Prostate and Urothelial Cancers

**DOI:** 10.3389/fonc.2020.00114

**Published:** 2020-02-07

**Authors:** Rohan Garje, Raju Kumar Vaddepally, Yousef Zakharia

**Affiliations:** ^1^Division of Hematology, Oncology, and Blood and Marrow Transplant, Holden Comprehensive Cancer Center, University of Iowa, Iowa City, IA, United States; ^2^Yuma Regional Medical Center, Yuma, AZ, United States

**Keywords:** prosate cancer, urothelial cancer (UC), PARP inhibitor, precision oncology, DNA damage repair genes

## Abstract

Poly(ADP-ribose) polymerase (PARP) inhibitors targeting DNA repair gene mutations have shown significant clinical benefit in patients with ovarian and breast cancers. In metastatic prostate cancers, the prevalence of DNA repair gene mutations is up to 20%, and early phase studies have shown clinical activity of PARP inhibitors. Numerous clinical trials with either PARP monotherapy or in combination with other therapeutic agents are ongoing in prostate cancer. In this comprehensive review, we provide the rationale, efficacy, and safety data of PARP inhibitors in prostate as well as urothelial cancers.

## Introduction

In the current era of precision medicine, the discovery of targeted agents has led to significant development in the armamentarium of cancer therapeutics (1–3). Many studies have identified new mechanisms to counter cancer cellular mechanisms (4). Targeting tumors with defects in DNA damage repair genes by inhibition of poly(ADP-ribose) polymerase (PARP) enzymes is one such breakthrough discovery (5). Multiple PARP inhibitors for the treatment of ovarian and breast cancers that harbor pathogenic *BRCA* mutations have been developed. PARP inhibitors are currently being explored in other cancers, including prostate, bladder, pancreas, and biliary tree (6).

## Parp Inhibitor Mechanism and the Concept of Synthetic Lethality

Cellular DNA is subject to continuous damage from intrinsic and extrinsic mutagens, which gives rise to single-strand (ss) and double-strand (ds) DNA breaks (7). DNA damage response pathways (DDR) ensure the conformity of the DNA sequence, and cells respond to this perception by arresting cell cycle progression and attempting repair (8). Depending on the type of DNA damage, several repair pathways such as base excision repair (BER), mismatch repair (MMR), and nucleotide excision repair (NER) aid in ssDNA breaks (9). For dsDNA breaks, there are two main mechanisms for DNA repair-homologous recombination (HR) and non-homologous end-joining (NHEJ)—where HR matches the original DNA in a seamless repair, and NHEJ introduces deletions (10).

PARP enzyme proteins play a vital role in DNA repair, promoting ss- and dsDNA repair (11–13). PARP-1 functions as a transcription modulator and regulates the oncogenes, tumor suppressor genes, and inflammatory genes involved in chromatin modulation and gene transcription (14, 15). One of the notable dsDNA break repair (DSBR) mechanisms is the HR repair (HRR) pathway, which facilitates seamless repair of dsDNA breaks. Genes involved in HRR include *BRCA2, BRCA1, PALB2*, and *ATM* (16, 17).

The concept of synthetic lethality applies when mutation or decreased expression of two genes results in cell death, whereas mutation of one gene alone leads to viability (18, 19). Synthetic lethality with PARP inhibitor is produced by conditional drug sensitivity in HRR-deficient cells. *BRCA1* and *BRCA2* are tumor suppressor genes, and defective tumors with loss of the copy of either gene are shown to be intrinsically sensitive to PARP inhibitors in both pre-clinical and clinical models (20, 21). Thus, this makes the loss of a gene essential for HRR to result in synthetic lethality from PARP inhibition, in which two pathway defects that alone are non-toxic but when combined become lethal (22).

## Parp Inhibitors in Prostate Cancer

Prostate cancer is the most common malignancy in men with an estimated incidence of 174,650 new cases in the United States in 2019 (23). The prevalence of mutations in the DNA repair genes involved in HRR in men with prostate cancer irrespective of age or family history is around 11–23%, with most common mutations noted in *BRCA2* (24–26). The other common mutated genes include *ATM, CHEK2, BRCA1, RAD51D, FANCA, CDK12*, and *PALB2*. These mutations are more prevalent in metastatic cancer than localized disease (24–26). Additionally, these mutations have been noted in high frequency with intraductal adenocarcinoma histology, lower PSA levels at diagnosis, and tumors with lymphovascular invasion (27). Current National Comprehensive Cancer Network (NCCN) guidelines recommend germline testing for HRR genes in patients with regional and metastatic prostate cancer (25). Multiple clinical trials are now evaluating the potential role of PARP inhibitors in metastatic prostate cancer, which has opened the door for targeted therapeutics (Tables 1, 2).

**Table 1:** Results of PARP inhibitors in prostate cancer.

**Study**	**Agent(s)**	**Cohort**	**Total enrollment**	**Response rate**	**Median rPFS (months)**	**Median OS (months)**
TOPARP A (28)	Olaparib 400 mg BID	mCRPC with prior chemotherapy and ≥1 NHA	49 (BM+: 16)	Entire cohort: 33% BM+: 88%	BM–: 2.7 BM+: 9.8	BM–: 7.5 BM+: 13.8
TOPARP B (29)	Olaparib 400 mg BID vs. olaparib 300 mg BID (randomized 1:1)	mCRPC with prior chemotherapy, NHA, and positive DDR gene aberrations	Ola 400: 49 Ola 300: 49	^*^Composite response:Ola 400: 54.3% of 46 evaluable Ola 300: 39.1% of 46 evaluable	Ola 400: 5.5 Ola 300: 5.4	Ola 400: 14.3 Ola 300: 10.1
PROfound (preliminary results) (30)	Olaparib 300 mg BID vs. pcNHA (randomized 2:1)	mCRPC with prior NHA, no chemotherapy, and selected for DDR gene aberrations	^†^Cohort A: Ola (162) vs. pcNHA (83) Cohort A+B: Ola (256) vs. pcNHA (131)	Cohort A: 33% vs. 2.3% Cohort A+B: 21.7% vs. 4.5%	Cohort A: 7.39 vs. 3.55 Cohort A+B: 5.82 vs. 3.52	Cohort A: 18.5 vs. 15.11 Cohort A+B: 17.51 vs. 14.26
Clarke et al. (31)	Abiraterone with olaparib 300 mg BID or placebo (randomized 1:1)	mCRPC with prior chemotherapy and no NHA; mixed cohort of HRR mutated and wild type	Abi+Ola: 71 Abi+placebo: 71	Abi+Ola: 27% Abi+placebo: 32% (33 and 38 patients had measurable disease in each cohort, respectively)	Abi+Ola: 13.8 Abi+placebo: 8.2	Abi+Ola: 22.7 Abi+placebo: 20.9 (HR 0.91; 95% CI 0.60–1.38); *p* = 0.66
TRITON2 (preliminary results) (32)	Rucaparib 600 mg BID	mCRPC with prior NHA, chemotherapy, and DDR gene aberrations	136	*BRCA 1/2*: 47.5% *ATM*: 0 *CDK12*: 0 ^‡^Other: 33.3%	Not reported	Not reported
GALAHAD (preliminary results) (33)	Niraparib 300 mg OD	mCRPC with prior NHA, chemotherapy, and DDR gene aberrations	81: *BRCA 1/2*, 46; ^§^*non-BRCA*, 35	*BRCA 1/2*: 41% *Non-BRCA*: 9%	*BRCA 1/2*: 8.2 *Non-BRCA*: 5.3	*BRCA 1/2*: 12.6 *Non-BRCA*: 14

BM, biomarkers for DNA damage repair genes; DDR, DNA damage repair; mCRPC, metastatic castration-resistant prostate cancer; rPFS, radiographic progression-free survival; OS, overall survival; pcNHA, physician choice novel hormonal agent (abiraterone or enzalutamide); Abi, abiraterone; Ola, olaparib, BID, twice daily; OD, once daily.

* *Composite response defined as any of the following outcomes: radiological response as per RECIST 1.1 with PCWG2 modification, decrease in PSA of ≥50% from baseline, and conversion of circulating tumor cell count (from ≥5 cells per 7.5 mL blood at baseline to <5 cells per 7.5 mL blood)*.

† *BRCA 1/2 or ATM. Cohort B: BARD1, BRIP1, CKD12, CHEK1, CHEK2, FANCL, PALB2, PPP2R2A, RAD51B, RAD51C, RAD51D, RAD54L*.

‡ *Includes FANCA, NBN, or PALB2, BRIP1, BRIP1/CHEK2, CDK12/CHEK2, CHEK2, RAD51, or RAD51B. In TRITON2, response was defined as complete or partial response per modified RECIST/PCWG3*.

§ *ATM, FANCA, PALB2, CHEK2, BRIP1, or HDAC2*.

**Table 2:** Summary of ongoing clinical trials evaluating PARP inhibitors as monotherapy or in combination with other therapeutic agents in prostate cancer.

**Study**	**Estimated enrollment**	**Design**	**Cohort**	**Agent(s)**	**Homologous recombination repair mutations**	**Primary endpoint(s)**
**OLAPARIB**						
KEYLYNK-010, NCT03834519	780	Phase III, randomized	mCRPC after one prior NHA and chemotherapy	Pembrolizumab and olaparib (300 mg BID) vs. abiraterone or enzalutamide	Unselected	OS2. rPFS
PROfound, NCT02987543	340	Phase III, randomized	mCRPC after one prior NHA	Olaparib (300 mg BID) vs. enzalutamide or abiraterone	Selected	rPFS
PROpel, NCT03732820	720	Phase III, randomized	First line treatment for mCRPC without prior NHA or chemotherapy	Olaparib (300 mg BID) and abiraterone vs. placebo and abiraterone	Unselected	rPFS
NCT03810105	32	Phase II, single arm	Biochemically recurrent nmCSPC	Olaparib (300 mg BID) and Durvalumab 1,500 mg IV monthly	Selected	Number of participants with undetectable PSA
LuPARP, NCT03874884	52	Phase I, single arm	mCRPC after any number of prior NHAs and chemotherapy	177Lu-PSMA + olaparib	Not available	DLTRecommended phase II dose
TRAP Trial, NCT03787680	47	Phase II, non-randomized	mCRPC with prior NHA	Olaparib and AZD6738 (ATR Inhibitor)	Selected	Rate of response (CR or PR)PSA response ≥50% decline
COMRADE, NCT03317392	112	Phase I/II, randomized	mCRPC with prior NHA and chemotherapy	Olaparib and Radium-223 vs. Radium-223	Not available	Maximum tolerated dose rPFS
NCT02893917	90	Phase II, randomized	mCRPC with at least one prior therapy	Olaparib and cediranib (VEGFR inhibitor) vs. olaparib	Not available	rPFS
**RUCAPARIB**						
TRITON 2, NCT02952534	360	Phase II, open label, non-randomized	mCRPC with prior 1–2 NHAs and taxane based chemotherapy	Rucaparib	Selected	ORR and PSA response
TRITON 3, NCT02975934	400	Phase III, randomized	mCRPC with 1 prior NHA and should not have received chemotherapy for CRPC	Rucaparib vs. physician's choice of therapy	Selected	Radiographic PFS
CheckMate 9KD, NCT03338790	330	Phase II, non-randomized	mCRPC	Nivolumab in combination with either rucaparib, docetaxel, or enzalutamide	Selected	ORRPSA response rate: ≥50% decline
NCT03572478	NA	Phase Ib/IIa	mCRPC with prior ≥1 NHA and chemotherapy	Rucaparib 600 mg PO BID and Nivolumab 480 mg IV q4w	Unselected	DLT
NCT03840200	NA	Phase Ib	mCRPC with prior NHA	Rucaparib and ipatasertib	Unselected	DLT, PSA response
PLATI-PARP NCT03442556	20	Phase II	mCRPC with prior NHA and chemotherapy	Rucaparib maintenance after 4 cycles of docetaxel and carboplatin chemotherapy	Selected	rPFS
**NIRAPARIB**						
GALAHAD, NCT02854436	301	Phase II, open label	mCRPC with prior NHA and chemotherapy	Niraparib 300 mg orally once daily	Selected	ORR
QUEST, NCT03431350	150	Phase Ib-II, multi-arm, non-randomized	mCRPC with prior NHA or chemotherapy depending on cohort	Niraparib 200 mg with either JNJ-63723283 (anti-PD-1 antibody) or Abiraterone	Both selected and unselected	Incidence of toxicities, ORR
MAGNITUDE, NCT03748641	1,000	Phase III, randomized	mCRPC without prior chemotherapy or NHA	Abiraterone with either Niraparib (200 mg OD) or placebo	Selected	Radiographic PFS
NiraRad, NCT03076203	NA	Phase Ib	mCRPC with atleast 1 NHA and with or without prior chemotherapy	Niraparib orally daily along with Radium 223 every 4 weeks for 6 courses	Unselected	DLTs
**TALAZOPARIB**						
NCT03148795 TALAPRO-1	100	Phase II, non-randomized	mCRPC with prior taxane based chemotherapy and at least 1 NHA	Talazoparib 1 mg OD	Selected for HRR positive	ORR
TALAPRO-2, NCT03395197	872	Phase III, randomized	First-line therapy for mCRPC	Enzalutamide with talazoparib (0.5 mg OD) or placebo	Part 1: safetyPart 2: both HRR positive and negative will be treated	Part 1: safetyPart 2: radiographic PFS
**PAMIPARIB**						
NCT03712930	100	Phase II, non-randomized	mCRPC with >1 NHA and >1 taxane-based chemotherapy	Pamiparib 60 mg PO BID	Selected	ORR and PSA response rate

*mCRPC, metastatic castration resistant prostate cancer; mCSPC, metastatic castration sensitive prostate cancer; NHA, novel hormonal agent; OS, overall survival; PFS, progression-free survival; ORR, objective response rate; HRR, homologous recombination repair; DLT, dose-limiting toxicity*.

## Olaparib Monotherapy

Olaparib is an inhibitor of PARP1, PARP2, and PARP3 enzymes involved in DNA repair and is currently approved for the treatment of ovarian and breast cancers. In the pivotal investigator-initiated, phase II Trial of PARP Inhibition in Prostate Cancer (TOPARP-A), the antitumor activity of olaparib 400 mg twice a day was evaluated in patients with metastatic, castration-resistant prostate cancer (CRPC) previously treated with chemotherapy and novel hormonal therapy (28). A response (defined as radiological response based on RECIST v1.1, or ≥ 50% decline in PSA or a reduction in the number of circulating tumor cells from ≥5/7.5 ml blood at baseline to <5/7.5 ml) was noted in 16 of 49 evaluable patients. Among the patients who had a response, 88% (14/16) harbored homozygous deletions and/or deleterious mutations in *BRCA 1/2, ATM*, Fanconi anemia genes, and *CHEK2*. Interestingly, 100% of the patients (7/7) with *BRCA2* mutation had a response. The median progression-free survival (PFS) and median overall survival in patients with genomic aberrations were 9.8 and 13.8 months, respectively (28). Myelosuppression and fatigue were the most common treatment-related adverse effects (28). It is important to note that a predominant number of patients (94%, *n* = 31) who did not harbor these deleterious mutations had no response to olaparib (28).

Based on TOPARP-A data, the multicenter randomized phase III clinical trial (PROfound study) evaluated the efficacy of olaparib (30). In this landmark trial, patients with metastatic CRPC who received prior novel hormonal therapy and harbored alterations in HRR genes were randomized in a 2:1 fashion to receive either olaparib (300 mg BID) or physician's choice of novel anti-androgen agents such as enzalutamide or abiraterone. Patients were enrolled in cohort A (*n* = 245) if the tumors harbored *BRCA1, BRCA2*, or *ATM* mutations and cohort B (*n* = 142) with other DNA repair gene alterations. In both cohorts, median PFS significantly improved with olaparib when compared to the novel hormonal therapy. Clinical benefit was more prominent in cohort A, where the median PFS was 7.39 months with olaparib and 3.55 months with novel hormonal therapy (HR 0.34; 95% CI 0.25–0.47; *p* < 0.0001). In cohort A, objective response rates were also better with olaparib (33.3%) when compared to enzalutamide/abiraterone (2.3%). The interim overall survival (OS) analysis in cohort A showed a median OS of 18.5 months with olaparib and 15.11 months with novel hormonal therapy; however, statistical significance was not reached at the time of data cutoff. The most common adverse events with olaparib therapy were anemia, nausea, decreased appetite, and fatigue. At the time of data cutoff, there were no reports of myelodysplastic syndrome or leukemia (30).

## Combination Studies With Olaparib in Prostate Cancer

Preclinical data have shown potential synergy of PARP inhibitors and androgen receptor antagonists irrespective of HRR status in prostate cancer (34). One preclinical study demonstrated that reduced levels of the tumor suppressor protein CCDC6 led to sensitization of cancer cells to PARP inhibitors (35). The turnover of CCDC6 protein is regulated by the de-ubiquitinase USP7, which also controls androgen receptor (AR) stability. The combination of USP7-inhibitors and PARP inhibitors, by affecting CCDC6 stability and HRR and accelerating AR/ARv7 turnover, may provide a novel therapeutic option in advanced prostate cancer.

In a randomized, placebo-controlled, and double-blind phase II clinical trial, Clarke et al. evaluated the efficacy of olaparib and abiraterone irrespective of HRR mutations in patients with metastatic CRPC (31). Of the 142 patients enrolled, 71 received olaparib, and abiraterone and 71 received placebo and abiraterone. HRR mutation status was not known in all patients. Radiological PFS was 13.8 months with olaparib and 8.2 months with placebo. Time to second progression or death was 23.3 months vs. 18.5 months, respectively. Median OS was 22.7 months in the olaparib arm and 20.9 months in the placebo arm (not statistically significant). However, grade ≥ 3 adverse events and serious adverse events were higher in the olaparib arm when compared to the placebo arm (54% vs. 28% and 34% vs. 18%, respectively). Four treatment-related deaths were noted in olaparib arm (1 each due to pneumonitis, ischemic stroke, cardiac failure, and mediastinitis) (31). To further evaluate this combination therapy, a multicenter, randomized phase III study (NCT03732820, PROpel study) is currently recruiting patients.

Cohort A of the phase Ib Keynote-365 clinical trial is evaluating the safety and antitumor activity of the combination of pembrolizumab (200 mg IV every 3 weeks) along with olaparib (400 mg orally twice daily). Preliminary results were presented at ASCO Genitourinary symposium in 2019 (36). In 41 patients with metastatic CRPC previously treated with chemotherapy and novel hormonal agent, the combination therapy had a median PFS per prostate cancer working group 3 (PCWG-3) RECIST criteria of 5 months and a median OS of 14 months. In this cohort, none of the patients had DNA repair gene defects as detected by either by Guardant360 ctDNA or whole exome sequencing of biopsy tissue. Per RECIST criteria, partial response was seen in 7% and stable disease was noted in 46%. The disease control rate of ≥6 months was seen in 32%. Responses were observed in both PD-L1+ (combined positive score ≥1) and PDL1– patients. The most common treatment-related adverse events were anemia, fatigue, and nausea. There was one treatment-related death. A phase III clinical trial evaluating this combination therapy is currently open for enrollment in unselected metastatic CRPC patients (NCT03834519, KEYLYNK-010 study).

Additional studies evaluating the safety and antitumor activity of olaparib in combination with radioligands such as radium 223 (COMRADE, NCT03317392) and 177Lu-PSMA (NCT03874884), cediranib (VEGFR inhibitor, NCT02893917) and AZD6738 (ATR inhibitor, NCT03787680) are ongoing (Table 2).

## Rucaparib

Rucaparib camsylate is an oral, highly selective small molecule inhibitor of PARP1, PARP2, and PARP3. It is currently FDA-approved for the treatment of recurrent epithelial ovarian, fallopian tube, and primary peritoneal cancers (37). Multiple studies are evaluating its role in prostate cancer.

The preliminary results of the industry-funded multicenter, phase II clinical trial (TRITON2) evaluating the antitumor activity of rucaparib were presented at the European Society for Medical Oncology (ESMO) Congress in 2019 (38). In this clinical trial, patients with metastatic CRPC who were previously treated with chemotherapy, androgen receptor antagonists, and had HRR genomic alterations (somatic or germline) were treated with rucaparib 600 mg orally twice daily. An objective response (defined as complete or partial response) of 47.5% (19/40) and a PSA response (≥50% decrease) rate of 53.6% (37/69) was seen in patients with *BRCA 1/*2 mutations. However, no objective responses were noted in patients with *ATM* or *CDK12* gene alterations. The most common grade ≥3 toxicities were anemia, fatigue, and elevated liver enzymes. No treatment-related deaths were reported. A randomized phase III clinical trial (TRITON3, NCT02975934) is currently comparing the efficacy of rucaparib to either novel hormonal therapy or docetaxel chemotherapy in meteastatic CRPC with HRR deficiency. Another pilot study of 30 patients (TRIUMP, NCT03413995) is evaluating the role of upfront rucaparib as an alternative to androgen deprivation therapy in patients with metastatic castration sensitive prostate cancer who have germline HRR mutations. The combination of rucaparib with the checkpoint inhibitor nivolumab is also currently being evaluated in a phase II clinical trial (CheckMate 9KD, NCT03338790).

## Niraparib

Niraparib is another PARP inhibitor currently being explored in the management of prostate cancer. It is a selective inhibitor of PARP1 and PARP2 enzymes involved in DNA repair with a long half-life of 36 h, permitting once-daily dosing. Additionally, niraparib traps PARP1 and PARP2 enzymes with higher potency when compared to other PARP inhibitors, and the trapped PARP-DNA complexes have been demonstrated to be more cytotoxic than unrepaired ssDNA breaks (37). It is the only PARP inhibitor approved by the FDA for maintenance treatment of patients with recurrent epithelial ovarian, fallopian tube, or primary peritoneal cancer irrespective of *BRCA* mutation status (38).

The preliminary results of the phase II, open-label, multicenter, and industry sponsored GALAHAD clinical trial (NCT02854436) were presented at the ESMO congress in 2019 (33). This study is evaluating the safety and efficacy of niraparib 300 mg once daily in patients with metastatic CRPC who have HRR gene mutations and were previously treated with taxane-based chemotherapy and at least one androgen receptor antagonist. To date, of the 81 patients with biallelic HRR gene alterations, 46 have *BRCA 1/2* and the rest have other HRR mutations (biallelic mutations in *ATM, FANCA, PALB2, CHEK2, BRIP1*, or *HDAC2*). In the *BRCA 1/2* cohort, the median radiographic PFS reported to date is 8.2 months, and median OS is 12.6 months. An objective response has been noted in 41% of patients. In the non-BRCA cohort, the median radiologic PFS to date is 5.3 months, and median OS is 14 months. The most common grade 3–4 adverse events are myelosuppression, asthenia, and back pain.

The combination of niraparib and abiraterone has also been studied and niraparib dosing of 200 mg once daily was deemed safe to study in further trials (39). Efficacy data or HRR status not yet been reported. A phase III randomized, blinded study of abiraterone with niraparib (200 mg once daily) or placebo is also currently enrolling patients (MAGNITUDE, NCT03748641). HRR gene mutation status will be used for randomization.

## Veliparib

Veliparib, a PARP1 and PARP2 inhibitor, was one of the earliest PARP inhibitors evaluated in combination with abiraterone in patients with metastatic CRPC (40). In a phase II multicenter study, 148 patients stratified by ETS status were randomly assigned to abiraterone alone or abiraterone with veliparib. The study was negative, with no difference between both arms either for PSA response or radiological response. On exploratory biomarker analysis, response rates and radiologic PFS were better in both arms in patients with HRR gene mutations when compared to HRR wild type. Currently, no further studies are planned with veliparib in prostate cancer.

## Talazoparib

Talazoparib is an inhibitor of PARP1 and PARP2 enzymes currently FDA-approved for germline *BRCA* mutated locally advanced, metastatic breast cancer. TALAPRO-1 and−2 studies are currently evaluating the efficacy of talazoparib in metastatic CRPC.

## Mechanism of Resistance to Parp Inhibitors

Results of ongoing studies with PARP inhibitors are encouraging, especially with better objective response rates and PFS data. However, despite good initial benefit, these responses are short-term, and patients eventually experience disease progression. It is crucial to understand resistance mechanisms, which will help formulate subsequent treatment strategies. One such mechanism is acquired *BRCA2* reversion mutations, where previously *BRCA-2*-deficient tumor cells are able to achieve *BRCA2* proficiency due to constant selection pressure of PARP inhibition (41). Serial sequencing of circulating cell-free (cf) DNA can help monitor these reversion mutations in a non-invasive manner. In addition to this mechanism, the loss of MLL3/4 complex protein and PTIP helps stabilize the replication fork and, in turn, protect DNA from degradation (42). It is also prudent to note the differential response to PARP inhibitors in tumors that harbor *BRCA* vs. *non-BRCA* HRR genomic aberrations. In a retrospective study of 23 patients with *BRCA 1/2* and *ATM* mutations, none of the 6 patients with *ATM* mutations responded to olaparib (43). In this study, the median PFS in patients with *BRCA* 1/2 was 12.3 months as compared to 2.4 months with *ATM* mutations. It is postulated that *ATM* is a DNA damage sensor rather than a mediator of DNA repair—a potential explanation for this differential response. Further studies are needed to understand the primary refractoriness and acquired resistance mechanisms to PARP inhibitors.

## Parp Inhibitors in Urothelial Cancer

Bladder cancer is the most common malignancy of urinary system, and urothelial histopathology is the most common subtype in the United States (23). Platinum-based chemotherapy and checkpoint inhibitors are the most common modalities of treatment for advanced, unresectable urothelial cancer. Recently, enfortumab vedotin was granted accelerated approval by the FDA in third-line setting (44). Additionally, for a subset of patients with fibroblast growth factor receptor (FGFR2/3) alterations, erdafitinib was also granted accelerated approval (45).

Molecular characterization of 412 tissue specimens with muscle-invasive bladder cancer in the cancer genome atlas (TCGA) program revealed the frequency of genomic aberrations in DNA repair genes such as *ATM, ERCC2, RAD51B* to be in the range of 2–14% (46). In another review of 81 muscle-invasive bladder cancer specimens, the somatic mutations in the *BRCA 1/2, PALB2, FANCD2, ERCC2, ATM* genes were in the range of 3.7–12.3% (47). Additionally, the presence of DDR and repair gene mutations was associated with improved response to platinum-based chemotherapy in metastatic urothelial cancer (48). These findings led to the fruition of several studies evaluating the efficacy of PARP inhibitors in metastatic urothelial carcinoma.

In a phase II, open-label, industry-sponsored study, patients with advanced urothelial cancer who progressed on one or two lines of systemic therapy were treated with rucaparib 600 mg twice daily (49). This study enrolled patients with both HRR deficient and proficient tumors. However, the study was terminated after preliminary review by an independent data monitoring committee did not show adequate objective response rate and met the criteria for study discontinuation. Additional studies evaluating the efficacy of PARP inhibitors in a cohort of urothelial cancers that is selected for HRR deficiency are ongoing (Table 3).

**Table 3:** Summary of ongoing clinical trials evaluating PARP inhibitors as monotherapy or in combination with other therapeutic agents in urothelial cancer.

**Clinical trial**	**Estimated enrollment**	**Design**	**Cohort**	**Prior treatment(s) allowed**	**Agent(s)**	**DNA damage repair mutations**	**Primary endpoint(s)**
**OLAPARIB**							
NCT03448718	30	Phase II, single arm	Metastatic urothelial cancer	Chemotherapy naïve, cisplatin ineligible, or progression on first line of treatment	Olaparib 300 mg PO BID	Selected	ORR
NCT03375307	60	Phase II, single arm	Metastatic urothelial cancer	Received 1 or 2 prior treatment regimens (platinum based chemotherapy or immunotherapy)	Olaparib	Selected	ORR
BISCAY, NCT02546661	NA	Phase I, multi-arm	Metastatic urothelial cancer	One prior platinum based therapy	Module B: olaparib with durvalumab IV every 4 weeks	Selected	Safety and tolerability
BAYOU, NCT03459846	152	Phase II, randomized, double blind, placebo-controlled	Metastatic urothelial cancer	First line, platinum ineligible	Olaparib (300 mg PO BID) or placebo in combination with durvalumab (1500 mg IV q4w)	Unselected	PFS
NEODURVARIB, NCT03534492	29	Phase I, single-arm, open-label	Neoadjuvant for MIBC prior to surgery	None	Olaparib 300 mg b.i.d. and durvalumab IV every 4 weeks for 2 months	Unselected	Pathologic complete response
**RUCAPARIB**							
ATLAS, NCT03397394	200	Phase II, single-arm	Metastatic urothelial cancer	Received 1 or 2 prior treatment regimens	Rucaparib	Unselected	ORR
ARIES, NCT03824704	139	Phase II, open-label; two cohorts	Metastatic urothelial cancer	Not eligible to receive cisplatin chemotherapy and have declined carboplatin chemotherapy or had disease progression during or after platinum-containing chemotherapy	Cohort B: urothelial cancer Oral rucaparib and IV nivolumab	Selected	ORR
SEASTAR, NCT03992131	NA	Phase Ib/II, open-label, multi-arm	Advanced/ metastatic urothelial carcinoma	1 prior line of standard therapy	Arm B: urothelial cancer Oral rucaparib and IV sacituzumab govitecan	Selected	Safety, DLT, and overall response rate
**NIRAPARIB**							
NCT03945084	77	Phase II, randomized	Advanced/metastatic urothelial carcinoma	After platinum-based chemotherapy	Maintenance niraparib after platinum-based chemotherapy	Unselected	PFS
MORPHEUS-mUC, NCT03869190	NA	Phase Ib/II, open-label, multicenter, randomized umbrella	Advanced/metastatic urothelial carcinoma	After platinum-based chemotherapy	Atezolizumab + niraparib	Unselected	ORR

*ORR, overall response rate; PFS, progression-free survival; DLT, dose-limiting toxicity*.

## Combination Strategies in Urothelial Cancer

In addition to enhancing sensitization to platinum chemotherapy, the presence of DNA repair gene aberrations is also associated with an increase in tumor mutation load and infiltration of lymphocytes in the tumor microenvironment (50, 51). Also, there is an activation of the stimulator of interferon genes (STING) pathway, which leads to enhanced antitumor immune response and PDL1 expression on cancer cells (52, 53). In a retrospective review of patients with urothelial cancer harboring known and unknown deleterious HRR gene mutations, monotherapy with PD1/PDL1 inhibitors showed higher response rates (54). Based on this observation, it is hypothesized that the combination of PARP inhibitors with PD1/PDL1 inhibitors in urothelial cancer can improve antitumor activity and clinical outcomes (55). The preliminary results of a multi-arm, biomarker-driven, adaptive study of durvalumab (anti-PDL1 antibody) in combination with either olaparib or an FGFR1-3 inhibitor (AZD4547) or a TORC 1 and 2 inhibitor (vistusertib) were presented at ESMO congress in 2019 (56). Of 391 screened patients, 54 (14%) had HRR genomic alterations and were treated with olaparib combination therapy. This group had a high tumor mutation burden and a confirmed objective response rate of 35.7%. The 6-month PFS rate was 42%, and 1 year OS rate was 54%. Grades 3/4 treatment-related adverse events were seen in 21.4%.

## Summary

In the management of advanced prostate cancer harboring DDR genomic aberrations, multiple PARP inhibitors are currently under study with encouraging results. This is a giant leap toward precision oncology for a cancer that over the past several decades was devoid of predictive therapeutic biomarkers. Similarly, multiple studies evaluating the safety and efficacy of PARP inhibitors in urothelial cancers are up and coming. Precise biomarkers that can accurately predict response to these agents is pivotal for the success of these agents as differential responses are noted in *BRCA* vs. *non-BRCA* mutations. Finally, it is prudent to explore the resistance mechanisms to PARP inhibitors by utilizing non-invasive tools such as cfDNA, as this would help the development of subsequent treatment strategies.

## Author Contributions

RG: project development and research, manuscript writing, and editing. RV: manuscript writing and editing. YZ: manuscript editing.

### Conflict of Interest

YZ: Advisory Board: Amgen, Roche Diagnostics, Novartis, Jansen, Eisai, Exelixis, Castle Bioscience, Array, Bayer, Pfizer, Clovis, EMD Serono. Grant/research support: institutional clinical trial support from NewLink Genetics, Pfizer, Exelixis, Eisai. DSMC: Jansen. The remaining authors declare that the research was conducted in the absence of any commercial or financial relationships that could be construed as a potential conflict of interest.
